# The effects of feeding rations that differ in neutral detergent fiber and starch within a day on the daily pattern of key rumen microbial populations

**DOI:** 10.3168/jds.2021-0099

**Published:** 2021-08-26

**Authors:** I.J. Salfer, C.E. Crawford, L.W. Rottman, K.J. Harvatine

**Affiliations:** 1 Department of Animal Science, University of Minnesota, St. Paul 55108; 2 Department of Animal Science, The Pennsylvania State University, University Park 16802

## Abstract

The effect of feeding a single TMR versus multiple rations across the day that differ in concentrations of neutral detergent fiber (NDF) and starch on the daily pattern of rumen microbial populations was characterized. Diets included a control total mixed ration (CON; 33.3% NDF), a low-fiber diet (LF; 29.6% NDF), and a high-fiber diet (HF; 34.8% NDF). Nine cannulated Holstein cows were assigned to 1 of 3 treatment sequences in a 3 × 3 Latin square design. Treatments included feeding CON ad libitum at 0900 h, feeding HF at 70% of daily offering at 0900 h and LF at 30% of daily offering at 2200 h (H/L), and feeding LF at 30% of daily offering at 0900 h and HF at 70% of daily offering at 1300 h (L/H). Rumen digesta was collected to represent every 3 h across the day, microbial DNA was extracted, and real-time quantitative PCR was used to determine the relative abundances of total bacteria, total fungi, total protozoa, *Butyrivibrio fibrisolvens*, *Butyrivibrio hungatei*, *Fibrobacter succinogenes*, *Megasphaera elsdenii*, *Prevotella bryantii*, *Ruminococcus albus*, *Selenomonas ruminantium*, and *Streptococcus bovis*. The relative abundances of total bacteria, total ciliated protozoa, *F. succinogenes*, *P. bryantii*, *R. albus*, *S. ruminantium*, and *Strep. bovis* were affected by time of day. Additionally, treatment affected the relative abundance of certain microbial groups at specific times of day. Notably, H/L treatment dramatically increased the relative abundances of *B. fibrisolvens*, *B. hungatei*, and *Strep. bovis* at 0900 h, by 2.5-, 5.4-, and 4.4-fold, respectively. Furthermore, the relative abundances of *B. hungatei* (3.9-fold), *M. elsdenii* (3.9-fold), *R. albus* (1.3-fold), *S. ruminantium* (1.3-fold), and *Strep. bovis* (4.5-fold) were greatly increased by L/H at 0900 h. At 0600 h, the relative abundance of *F. succinogenes* was 58% greater in L/H than H/L and the relative abundance of *P. bryantii* was 49% greater in H/L than L/H. Results suggest that there is a daily pattern of selected microbial populations that is altered by feeding rations that differ in NDF and starch within a day, with the greatest difference occurring before morning feeding.

## Highlights

• Rumen microbial relative abundance follows a daily pattern.
• Feeding 2 diets differing in starch and neutral detergent fiber (NDF) modifies microbial daily pattern.
• *Streptococcus bovis* and *Butyrivibrio* peaked before feeding of a high-NDF diet in the morning.
• *Ruminococcus albus, Selenomonas ruminantium*, and *Fibrobacter succinogenes* increased before feeding a low NDF diet.

## Graphical Abstract

**Figure:**
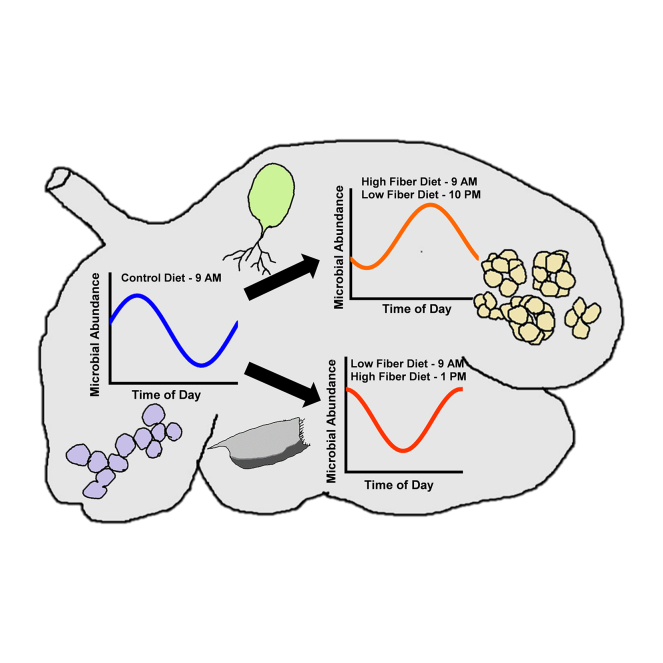
**Summary:** The timing of feeding of starch and fiber affects daily patterns of rumen pH, nutrient digestion, and milk production but there is little information on the effect on daily patterns of rumen microbial populations. This study measured the relative abundance of rumen bacteria, protozoa, fungi and bacterial species in response to feeding diets that altered the concentration of fiber and starch to cows across the day. The results show that that relative abundance of microbial species is dynamic across the day and that their daily patterns are affected by the timing of feeding starch and fiber.

Cattle naturally exhibit a crepuscular pattern of feed intake and traditionally consume greater amounts of feed in the morning and evening (Albright, 1993). In dairy cows, milking and the delivery of fresh feed act as strong stimuli for intake, and periods of high intake occur after feed delivery and milking and during the afternoon and early evening (DeVries et al., 2003). Most dairy farms currently feed a TMR once or twice daily, which is expected to deliver a consistent concentration of nutrients per meal. However, because of cows' daily feeding patterns, there is a large change in the amount of feed entering the rumen across the day, with 3 to 8 times more fermentable substrate entering the rumen during high-intake periods (Ying et al., 2015). These observations stand in direct opposition to the assumption of a rumen steady state that is used in most nutritional models (Fox et al., 2004).

Further evidence of daily fermentation dynamics is provided by diurnal observations of rumen pH. Rumen pH typically peaks just before feeding and declines to reach a nadir about 10 h later during the high intake period of the day (Yang and Beauchemin, 2006; Salfer et al., 2018). The daily pattern of rumen pH is opposite that of *trans*-10 C18:1 fatty acids in the rumen, which are associated with biohydrogenation-induced milk fat depression (Ying et al., 2015). We previously reported that the daily pattern of rumen pH was altered by feeding rations that differed in NDF and starch concentrations within a day (Ying et al., 2015). Moreover, total VFA, acetate, and propionate concentrations also follow a daily rhythm, with all reaching a nadir at approximately 0900 h (Ying et al., 2015). Rumen redox potential (Richter et al., 2010) and methane production follow daily rhythms that are responsive to feeding (Crompton et al., 2011; Zhou et al., 2011). Finally, total rumen volume and DM and NDF pool sizes are modified by diet and follow a daily rhythm, suggesting that rumen outflow also follows a daily rhythm (Gasa et al., 1991; Ying et al., 2015).

The daily patterns of nutrient intake, nutrient digestion, rumen pH, and rumen turnover may cause—or be caused by—changes in rumen microbial abundance and composition across the day. Bryant and Robinson (1961) reported a daily pattern of bacterial abundance quantified as total bacterial colony counts in the rumen, and the pattern differed between grain- and hay-based diets. The abundance of bacteriophage also displays a cosine-shaped daily pattern with a peak about 8 to 10 h post-feeding (Klieve and Swain, 1993). Furthermore, apparent microbial protein synthesis follows a daily pattern in sheep, peaking approximately 8 h after feeding (Walker and Nader, 1970). Recent research has suggested that gut microbes of mice undergo daily oscillations and may be under the control of circadian rhythms (Liang and FitzGerald, 2017). Moreover, a molecular circadian mechanism has been established in cyanobacteria, demonstrating the possibility of true circadian rhythms in prokaryotes (Pattanayak and Rust, 2014). However, to our knowledge, no experiments have examined daily patterns of specific microbial species in dairy cows.

We previously proposed that changing diet composition across the day could create a more stable rumen environment (Rottman et al., 2015). Briefly, feeding a higher NDF and lower starch diet during the high-intake period of the day and a lower NDF and higher starch diet during the lower-intake period during the night was expected to create a more consistent amount of starch entering the rumen. In contrast, feeding a low-NDF and high-starch diet first during the high-intake period of the day was designed to exacerbate changes across the day.

The objective of the current experiment was to examine the effect of feeding rations that differ in NDF and starch within a day on daily patterns of total rumen bacteria, protozoa, fungi, and bacterial species with well-defined niches within the rumen. We hypothesize that modifying the timing of fiber and starch feeding will shift the daily patterns of microbial relative abundance, such that fiber-utilizing species and anaerobic fungi will peak within 4 h of feeding the high-fiber diet and that starch-utilizing species will peak within 4 h of feeding the high-starch diet. Furthermore, the relative abundance of bacteria is expected to be stabilized when the high-fiber diet is fed in the morning due to the slower growth of fibrolytic bacterial species. The effects on feeding behavior, milk yield, FA profile, and plasma hormone and metabolite concentrations (Rottman et al., 2015) and daily changes in rumen digesta nutrient concentration and pool size including starch, NDF, and individual FA, rumen pH, and VFA (Ying et al., 2015) have been previously reported.

Experimental procedures were approved by the Penn State University Institutional Animal Care and Use Committee. Nine multiparous lactating Holstein cows (158 ± 48 DIM, mean ± SD) from the Pennsylvania State University Dairy Research and Teaching Center were randomly assigned to 1 of 3 treatment sequences in a 3 × 3 Latin square design with 23-d periods (n = 9 per sequence), as described by Rottman et al. (2015). Sample size was determined for the original experiment based on >80% power of observing a *P* < 0.05 difference in milk yield based on the variance observed in previous experiments. Randomization was conducted using the rand() function of Excel (Microsoft Corp.), generating random numbers corresponding to each stall and ranking the random numbers numerically to match stalls with treatment sequences. Treatment sequences were balanced to minimize carryover effects.

Cows were housed in tiestalls with a 19-h light phase and a 5-h dark phase from 0000 to 0500 h. Cows were milked twice per day at 0700 and 1700 h. Three rations were fed: a control complete TMR (**CON**; 33.1% NDF), a low-fiber diet (**LF**; 29.6% NDF), and a high-fiber diet (**HF**; 34.8% NDF). Dietary NDF level was modified by substituting corn grain for forage. Diets were balanced so that a 7:3 ratio of HF:LF offered the same nutrient composition as the CON diet. Treatments included feeding CON at 0900 h, feeding HF at 70% of daily offering at 0900 h and LF at 30% of daily offering at 2200 h (**H/L**), and feeding LF at 30% of daily offering at 0900 h and HF at 70% of daily offering at 1300 h (**L/H**). All cows were fed at 110% of expected daily intake and orts were removed before feed delivery.

Rumen digesta were sampled every 9 h from d 15 to 17 of each period to represent every 3 h of the day (0000, 0300, 0600, 0900, 1200, 1500, 1800, and 2100 h). Manual grab samples of whole digesta were collected from 6 regions of the rumen (cranial dorsal, cranial ventral, central, caudal, dorsal, and caudal ventral). Digesta were composited, subsampled (approximately 250 g), and stored at −20°C before analysis. Samples were lyophilized (VirTis Ultra 35-XL, SP Industries) and ground using a consumer-grade coffee grinder (model 90 335; Hamilton Beach Brands Inc.) for 60 s. DNA was extracted using a commercially available extraction kit (E.Z.N.A. Stool DNA Kit; Omega Bio-Tek) with modifications similar to those in Yu and Morrison (2004), as described by Rico et al. (2015). Concentration and purity of DNA were determined by spectrophotometry at 260 and 280 nm (NanoDrop ND-1000, NanoDrop Technologies). All samples were diluted to 100 µg/dL before downstream analysis. The relative abundances of total bacteria, *Butyrivibrio fibrisolvens*, *Butyrivibrio hungatei*, *Fibrobacter succinogenes*, *Megasphaera elsdenii*, *Prevotella bryantii*, *Prevotella ruminicola*, *Ruminococcus albus*, *Selenomonas ruminantium*, and *Streptococcus bovis* were determined using real-time quantitative PCR (**qPCR**) with previously designed primers (Table 1) targeting the 16S rRNA gene according to Rico et al. (2015). Total anaerobic fungi and total ciliate protozoa were quantified by qPCR of the 18S rRNA gene using primers described by Sylvester et al. (2004) and Denman and McSweeney (2006), respectively (Table 1). These organisms were chosen because of their established metabolic niches in the rumen. Quantification was performed using the relative standard curve method with an Applied Biosystems 7900HT Fast Real-Time PCR system (Applied Biosystems Inc.) and SYBR Green reporting dye (PerfeCTa Supermix with ROX, Quanta Biosciences). Primer specificity was evaluated using melting curve analysis, and primer efficiency (*E*) was calculated from the standard curve as *E* = 10^−1/slope^. Efficiency values ranged from 84 to 126% (98.0% ± 2.1%; mean ± SEM). Relative abundance of bacteria, anaerobic fungi, and ciliate protozoa and individual bacterial species were calculated relative to a standard curve with 2-fold dilutions included in duplicate on each qPCR plate. Relative abundance of individual bacterial species was expressed as a percentage of total bacteria.

**Table 1:** List of primers used to quantify microbial groups using quantitative real-time PCR

Organism	Primer (top: forward; bottom: reverse)^1^	T_a_^2^	Reference
Bacteria	CGGCAACGAGCGCAACCC	60	Denman and McSweeney (2006)
	CCATTGTAGCACGTGTGTAGCC		
Ciliate protozoa	GCTTTCGWTGGTAGTGTATT	55	Sylvester et al. (2004)
	CTTGCCCTCYAATCGTWCT		
Fungi	GAGGAAGTAAAAGTCGTAACAAGGTTTC	60	Denman and McSweeney (2006)
	CAAATTCACAAAGGGTAGGATGATT		
*Butyrivibrio fibrisolvens*	GCCTCAGCGTCAGTAATCG	65	Shingfield et al. (2012)
	GGAGCGTAGGCGGTTTTAC		
*Butyrivibrio hungatei*	AGGGTAATGCCTGTAGCTC	55	Shingfield et al. (2012)
	TCACCCTCGCGGGAT		
*Fibrobacter succinogenes*	GCGGGTAGCAAACAGGATTAGA	60	Stevenson and Weimer (2007)
	CCCCCGGACACCCAGTAT		
*Megasphaera elsdenii*	GACCGAAACTGCGATGCTAGA	60	Ouwerkerk et al. (2002)
	CGCCTCAGCGTCAGTTGTC		
*Prevotella bryantii*	AGCGCAGGCCGTTTGG	61	Stevenson and Weimer (2007)
	GCTTCCTGTGCACTCAAGTCTGAC		
*Ruminococcus albus*	CCCTAAAAGCAGTCTTAGTTCG	61	Wang et al. (1997)
	CCTCCTTGCGGTTAGAACA		
*Selenomonas ruminantium*	CAATAAGCATTCCGCCTGGG	61	Stevenson and Weimer (2007)
	TTCACTCAATGTCAAGCCCTGG		
*Streptococcus bovis*	TTCCTAGAGATAGGAAGTTTCTTCGG	60	Stevenson and Weimer (2007)
	ATGATGGCAACTAACAATAGGGGT		

1 Selected forward and reverse primers for validated for genes encoding 16S ribosomal RNA (bacterial species and fungi), and for the 18S rRNA gene (protozoa).

2 T_a_ = annealing temperature (°C).

All data were analyzed as repeated measures using the MIXED procedure of SAS 9.4 (SAS Institute Inc.). The model included the fixed effects of treatment, time, and the interaction of treatment and time, as well as the random effects of cow, period, and qPCR plate to remove the effect of technical variation among plates (n = 3 cows/plate). Time was included as a repeated variable within the model. The autoregressive covariance structure was chosen over the heterogeneous structure based on Bayesian information criterion and convergence criteria, and denominator degrees of freedom were adjusted using the Kenward-Roger method. Preplanned contrasts were used to test differences between CON and H/L and between H/L and L/H at each time point. Statistical significance was declared at *P* < 0.05 and trends noted at 0.05 < *P* < 0.10. Heteroscedasticity was determined by generating a histogram and normal Q-Q plot of residuals in PROC MIXED. High-resolution figures were generated using an add-in for Microsoft Excel (Kraus, 2014).

In a previously published paper, we observed that both H/L and L/H decreased total DMI relative to CON, which coincided with a decrease in feeding time (Rottman et al., 2015). Moreover, milk and milk fat yields were greater for H/L than L/H. Additionally, the eating rate post-feeding was increased in L/H relative to H/L, which was associated with increased rumen pH, propionate concentrations, and concentrations of *trans*-10 C18:1 before feeding (Ying et al., 2015).

In the current analysis, the relative abundance of total bacteria was affected by time of day, suggesting a daily pattern (*P* = 0.005; Figure 1A). Bacterial abundance was greatest in the morning, similar to previous results by Bryant and Robinson (1961). Neither average bacterial abundance (*P* = 0.80) nor the daily pattern (*P* = 0.69) was affected by treatment. The relative abundance of ciliate protozoa was similarly affected by time of day (*P* < 0.0001) but not treatment (*P* = 0.26), nor was there a treatment by time interaction (*P* = 0.26; Figure 1B). Ciliate protozoa abundance peaked at 0600 h in L/H, at 0900 h in CON, and at 1200 h in H/L. Our results coincide with those observed by Michalowski and Muszyński (1978), who similarly detected variation in protozoal abundance across the day, with maximum abundance occurring immediately before feeding. Based on our results, it appears that the time of starch feeding is more closely associated with increasing protozoal abundance, which agrees with previous research suggesting that starch is a more potent stimulus of protozoal growth than fiber (Ishaq et al., 2017). Although protozoal reproduction is generally slower than bacterial cell division, certain species of *Entodinium* protozoa can replicate within 15 min, allowing for changes in the population throughout the day (Williams and Coleman, 1997). Notably, protozoa undergo chemotaxis toward areas of the rumen with greater nutrient density (Firkins et al., 2020). Even though we analyzed composite samples from all regions of the rumen, the observed differences in protozoal numbers might be partly due to migration of protozoa to different regions of the rumen.

**Figure 1:**
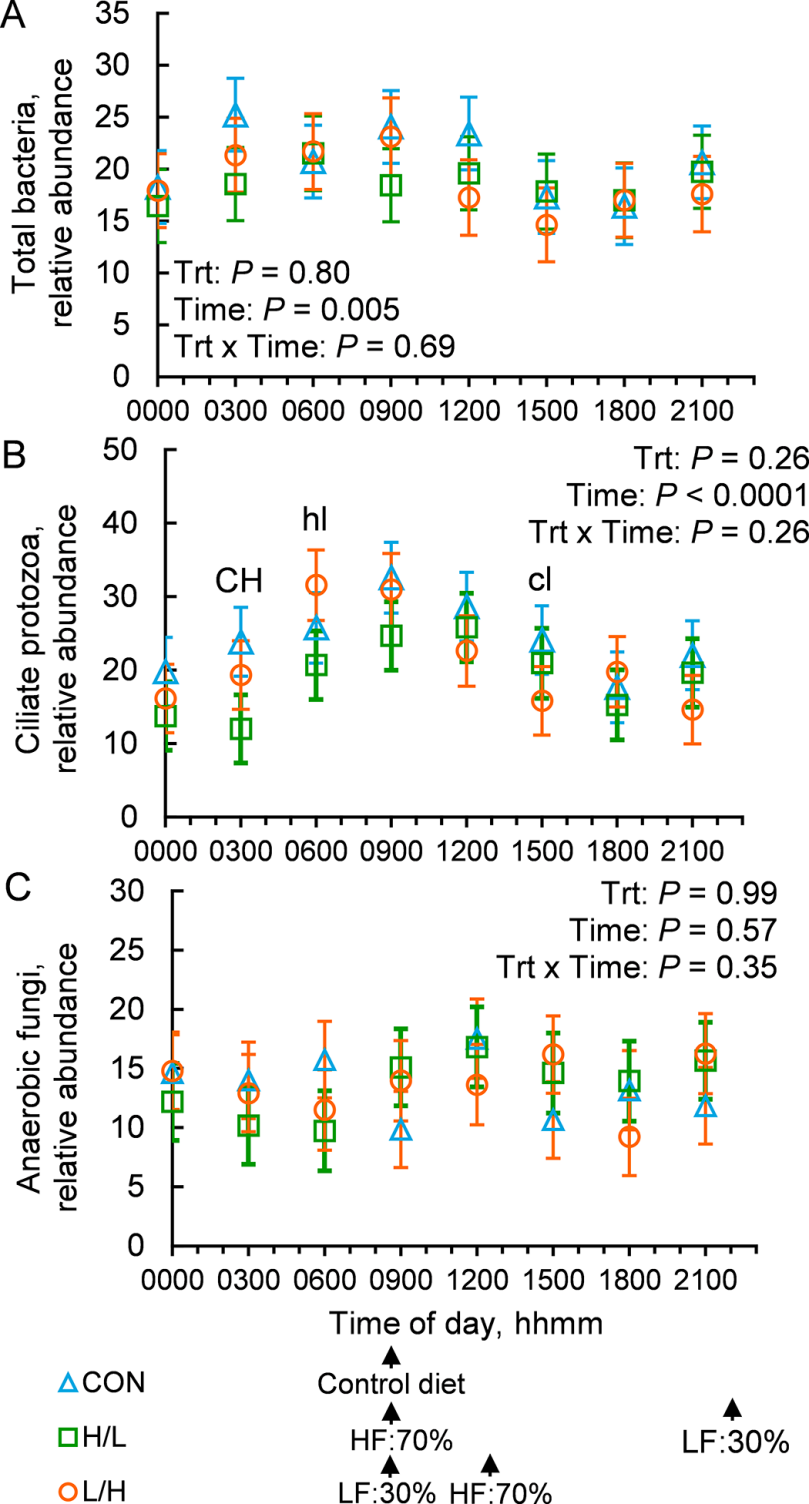
Effects of feeding rations that differ in NDF and starch within a day on relative abundances of (A) total bacteria, (B) ciliate protozoa, and (C) anaerobic fungi in the rumen across the day. Rations included a control complete TMR (33.1% NDF), a low-fiber diet (LF; 29.6% NDF), and a high-fiber diet (HF; 34.8% NDF). Treatments included feeding the control diet at 0900 h (CON), feeding HF at 70% of daily offering at 0900 h and LF at 30% of daily offering at 2200 h (H/L), and feeding the LF at 30% of daily offering at 0900 h and HF at 70% of daily offering at 1300 h (L/H). Treatment means with SEM bars and the effects of treatment (Trt), time, and their interaction are shown. Preplanned contrasts were used to test differences between CON and H/L and between H/L and L/H at each time point. Differences at individual time points are indicated with letters: C, H, and L denote CON, H/L, and L/H, respectively. Uppercase letters above time points indicate differences between denoted treatments (*P* < 0.05), and lowercase letters above time points indicate a tendency for difference between denoted treatments (0.05 ≤ *P* < 0.10).

The abundance of anaerobic fungi was not affected by treatment (*P* = 0.99) or time of day (*P* = 0.57), and there was no interaction of treatment and time of day (*P* = 0.35; Figure 1C). Results disagree with previous data that suggested a large increase in population density of fungi 1 h after feeding; however, those results were obtained using culture-based techniques, which may be biased by the cultivability of fungi species (Orpin and Joblin, 1997). Moreover, our results included total rumen contents, including fungal cells attached to plant cell walls within the solids phase of the rumen, which may have resulted in a more complete measurement of their populations. Rumen fungi have been purported to replicate only 2 to 4 times per day and, therefore, would have limited ability to change their population density greatly throughout a day (Orpin, 1975).

*Fibrobacter succinogenes* and *R. albus* are 2 of the best characterized cellulose-degrading bacterial species present in the rumen. Relative abundance of *F. succinogenes* differed across the day (*P* < 0.0001) but was not affected by treatment (*P* = 0.14) or the interaction of treatment and time (*P* = 0.74; Figure 2C). However, analysis at the 0600 h time point showed an increase in *F. succinogenes* under the L/H feeding schedule relative to H/L and a tendency for an increase relative to CON. One potential reason for the high relative abundance of *F. succinogenes* bacteria before feeding is the slow rate of fiber fermentation, which may lead to these organisms remaining in the rumen to continue to digest fiber, whereas more rapidly degraded nutrients like starch and amino acids may have been depleted at this time. The relative abundance of *R. albus* was affected by time (*P* < 0.0001) and there tended to be a treatment by time interaction (*P* = 0.09; Figure 2F). At the 0900 h time point, L/H increased *R. albus* abundance (*P* = 0.01), whereas H/L tended to decrease its abundance (*P* = 0.08). These results contradict our hypothesis that fiber-degrading bacterial species would be elevated after the high-fiber diet was fed, and instead suggest that growth of these species occurs closer to the time of feeding a high-starch, low-fiber diet. This outcome may be due to interrelationships with other microbial species or may be due to a several-hour delay in growth after feeding the high-fiber diet. *Butyrivibrio fibrisolvens*, which utilizes a diversity of structural and nonstructural carbohydrates, including cellulose and hemicellulose, was not affected by treatment (*P* = 0.29), time (*P* = 0.20), or their interaction (*P* = 0.29). However, when the 0900 h data were examined independently, H/L increased *B. fibrisolvens* abundance compared with CON (*P* = 0.0001) and L/H (*P* = 0.002; Figure 2A).

**Figure 2:**
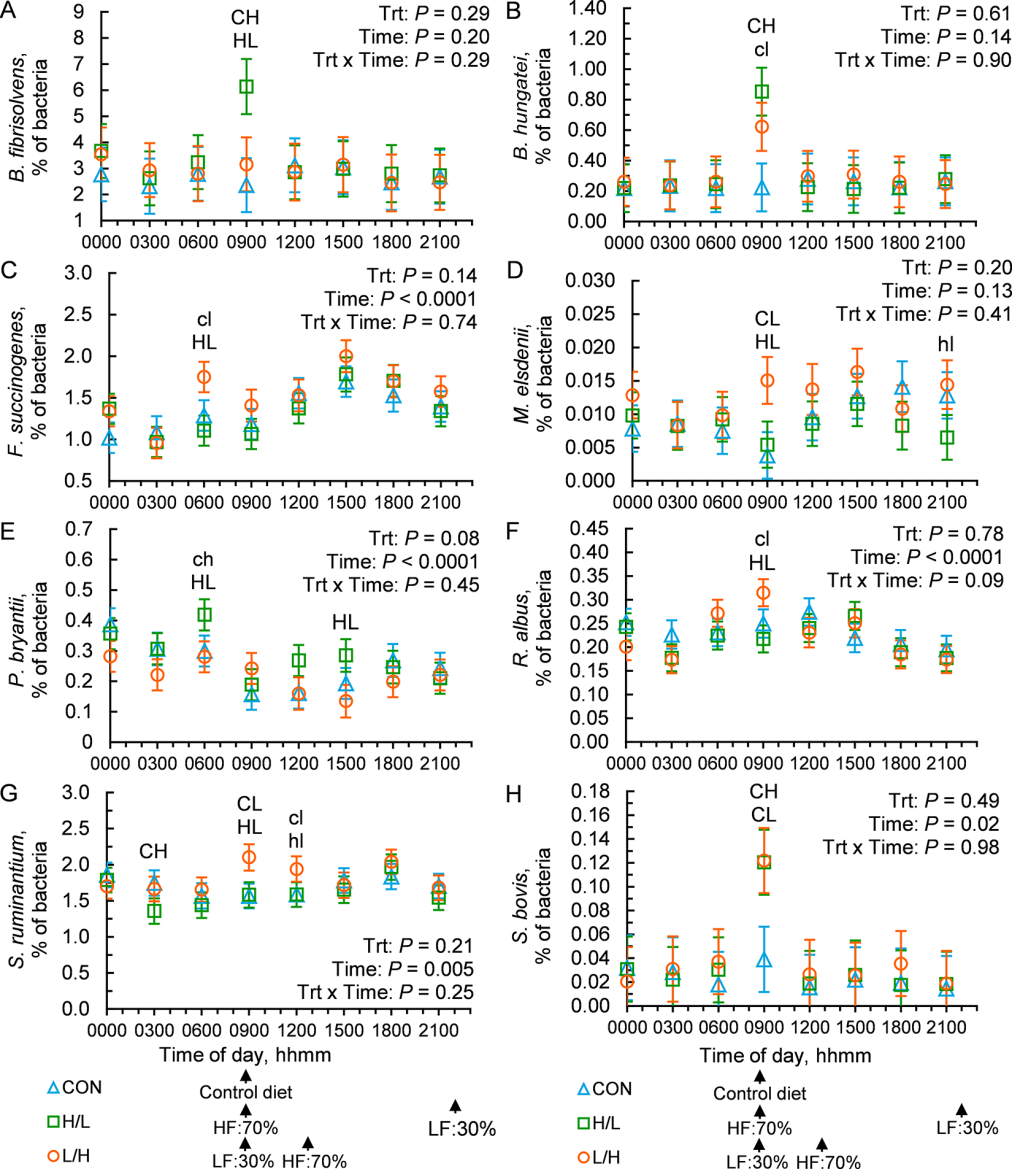
Effects of feeding rations that differ in NDF and starch within a day on relative abundances of prominent bacterial species in the rumen across the day. Bacterial species include (A) *Butyrivibrio fibrisolvens,* (B) *Butyrivibrio hungatei*, (C) *Fibrobacter succinogenes*, (D) *Megasphaera elsdenii*, (E) *Prevotella bryantii*, (F) *Ruminococcus albus*, (G) *Selenomonas ruminantium*, (H) *Streptococcus bovis.* Rations included a control complete TMR (33.1% NDF), a low-fiber diet (LF; 29.6% NDF), and a high-fiber diet (HF; 34.8% NDF). Treatments included feeding the control diet at 0900 h (CON), feeding HF at 70% of daily offering at 0900 h and LF at 30% of daily offering at 2200 h (H/L), and feeding the LF at 30% of daily offering at 0900 h and HF at 70% of daily offering at 1300 h (L/H). Treatment means with SEM bars and the effects of treatment (Trt), time, and their interaction are shown. Preplanned contrasts were used to test difference between CON and H/L and between H/L and L/H at each time point. Differences at individual time points are presented as letters: C, H, and L denote CON, H/L, and L/H, respectively. Uppercase letters above time points indicate differences between denoted treatments (*P* < 0.05), and lowercase letters above time points indicate a tendency for difference between denoted treatments (0.05 ≤ *P* < 0.10).

Notable nonstructural carbohydrate-degrading bacteria in the rumen include *Strep*. *bovis*, *S. ruminantium*, and *P. bryantii* (Russell, 2002). *Streptococcus bovis*, a rapid degrader of starch and a major producer of ruminal lactate, was affected by time (*P* = 0.02) but not treatment, and there was no treatment by time interaction (Figure 2H). At the 0900 h time point, the abundance of *Strep. bovis* was increased ~200% by both H/L (*P* = 0.04) and L/H (*P* = 0.03), suggesting that compared with feeding a single TMR, feeding 2 diets that differ in starch and fiber concentration cause a dramatic increase in *Strep. bovis* during initial feeding time, regardless of the timing of the 2 diets. *Selenomonas ruminantium*, which can utilize oligosaccharides, glucose, and lactate as substrates, also differed across the day (*P* = 0.005) but was not affected by treatment or the interaction of treatment and time (Cotta and Whitehead, 1998; Figure 2G). However, at the 0900 h time point, the L/H treatment increased abundance of *S. ruminantium* relative to CON and H/L (*P* = 0.01) and tended to increase its relative abundance at the 1200 h time point (*P* = 0.07). Moreover, H/L decreased *S. ruminantium* relative to CON at 0300 h (*P* = 0.05). Results suggest that *S. ruminantium* growth is stimulated within the first 6 h after feeding a high-starch diet, coinciding with its primary niche as a fermenter of nonstructural carbohydrates. The relative abundance of *P. bryantii* tended to be altered by treatment (*P* = 0.08) and was affected by time (*P* < 0.0001) but was not affected by the interaction of treatment and time (*P* = 0.45; Figure 2E). At 0600 h, H/L increased *P. bryantii* relative to L/H (*P* = 0.04) and tended to increase *P. bryantii* relative to CON (*P* = 0.45).

*Megasphaera elsdenii* and *B. hungatei* are 2 species that are important utilizers of rumen lactate and play a role in biohydrogenation of fatty acids. Neither treatment, time of day, or their interaction affected the relative abundance of *Megasphaera elsdenii* (Figure 2D). However, when the 0900 h time point was investigated independently, L/H increased abundance of *M. elsdenii* compared with CON (*P* = 0.01) and H/L (*P* = 0.02). The L/H treatment also tended to increase *M. elsdenii* relative abundance compared with H/L at the 2100 h time point (*P* = 0.05). Similarly, the relative abundance of *B. hungatei* was not affected by treatment (*P* = 0.61), time (*P* = 0.14), or the interaction of treatment and time (*P* = 0.90; Figure 2B). Specifically at 0900 h, H/L increased (*P* = 0.005) and L/H tended to increase (*P* = 0.07) *B. hungatei* relative abundance compared with CON.

Overall, the results of this experiment demonstrate that the abundance of microbial species is dynamic across the day and that their daily patterns are affected by the timing of feeding starch and fiber. Most notably, 2 species that rapidly ferment nonstructural carbohydrates, *Strep. bovis* and *B. hungatei*, rapidly increased during the first 3 h after the first daily feeding when the 2 diets differing in starch and fiber were fed, regardless of the order in which the 2 diets were fed. This may indicate that feeding rations that differ in composition over the day modifies microbial responsiveness and increases dynamic responses to feeding. Contrary to our hypothesis, the dynamics of individual microbial species abundance did not directly correspond to intake of nutrients that are known substrates for their growth, with prominent fiber-degrading bacteria increasing after high-starch diets were fed. It is likely that more complex dynamics were related to cross-feeding. Moreover, many of the microbial species examined may have niches beyond their traditionally assumed roles in the rumen. Future experiments with a broader scope, focusing on the complete rumen microbiome and metabolome, would provide additional insights into the daily patterns of rumen microbes and their relationship to nutrient intake.
